# Analgesic effects and pharmacologic mechanisms of the *Gelsemium* alkaloid koumine on a rat model of postoperative pain

**DOI:** 10.1038/s41598-017-14714-0

**Published:** 2017-10-27

**Authors:** Bo-Jun Xiong, Ying Xu, Gui-Lin Jin, Ming Liu, Jian Yang, Chang-Xi Yu

**Affiliations:** 1 Department of Pharmacology and College of Pharmacy, Fujian Medical University, Fuzhou, 350122 Fujian, People’s Republic of China; 2 Fujian Key Laboratory of Natural Medicine Pharmacology, College of Pharmacy, Fujian Medical University, Fuzhou, 350122 Fujian, People’s Republic of China

## Abstract

Postoperative pain (POP) of various durations is a common complication of surgical procedures. POP is caused by nerve damage and inflammatory responses that are difficult to treat. The neuroinflammation-glia-steroid network is known to be important in POP. It has been reported that the *Gelsemium* alkaloid koumine possesses analgesic, anti-inflammatory and neurosteroid modulating activities. This study was undertaken to test the analgesic effects of koumine against POP and explore the underlying pharmacologic mechanisms. Our results showed that microglia and astroglia were activated in the spinal dorsal horn post-incision, along with an increase of proinflammatory cytokines (interleukin 1β, interleukin 6, and tumor necrosis factor α). Both subcutaneous and intrathecal (i.t.) koumine treatment after incision significantly prevented mechanical allodynia and thermal hyperalgesia, inhibited microglial and astroglial activation, and suppressed expression of proinflammatory cytokines. Moreover, the analgesic effects of koumine were antagonized by i.t. administration of translocator protein (18 kDa) (TSPO) antagonist PK11195 and GABA_A_ receptor antagonist bicuculline. Together, koumine prevented mechanical allodynia and thermal hyperalgesia caused by POP. The pharmacologic mechanism of koumine-mediated analgesia might involve inhibition of spinal neuroinflammation and activation of TSPO. These data suggested that koumine might be a potential pharmacotherapy for the management of POP.

## Introduction

Postoperative pain (POP) of varying duration is extremely common after surgery. Long-lasting, life-changing painful sequelae caused by surgical injury have been long recognized as a major clinical problem^1^. POP is difficult to treat and, in many cases, prevents the return to normal activities of life. Evidence suggests that 86% of patients who undergo surgical experience pain and 75% of those with moderate to extreme pain^2^. Despite there has been an increased emphasis on the need for effective management of pain, POP continues to be inadequately treated. This represents a major public health and economic concern.

The mechanisms that determine the duration of POP are poorly understood. The course of POP is attributed to both primary hyperalgesia at the site of injury and secondary hyperalgesia at regions not directly affected by the surgical procedure^3^. Primary hyperalgesia from surgical incisions and other manipulations invariably causes some measure of nerve damage and inflammatory response that, in some cases, lead to the development of lasting forms of secondary hyperalgesia, such as neuropathic pain. This longer duration pain results from “central sensitization” in the spinal cord (SC) and brain (i.e. neuropathy) following peripheral injury. Experimental animal models (mostly in rodents), such as plantar incision, that mimic the transition from primary hyperalgesia to secondary hyperalgesia and neuropathy are important for studying the underlying causes of POP and evaluating novel therapies^4–6^. In these models, a surgical incision through the skin and muscles of the foot (or back) lead to 3–5 days of acute post-incisional pain, which is then manifest in different severities of secondary hyperalgesia. Studies using these experimental frameworks have advanced knowledge of the pathophysiologic processes that cause the transition from short-lived acute pain to pathologic chronic pain. For example, they have revealed a vital role for inflammatory mediators and glial cell activation in inducing nociceptor sensitization that leads to POP^7^. Moreover, local production of neurosteroids in glia cells is known to confer neuroprotection in central nervous system (CNS) inflammatory pain^8^ and translocator protein (18 KDa) (TSPO) is thought as the main target that could efficiently stimulate neurosteroidogenesis^9^. Among the neurosteroids, allopregnanolone (AP) has been broadly exploited since it executes analgesic effect through positive allosteric modulation of γ-aminobutyric acid type A (GABA_A_) receptor^10^. As a result, there has recently been increased focus in POP research on the neuroinflammation-glia-steroid network^7,8,11–13^.

*Gelsemium*, a genus of the family Loganiaceae, has long been used in Chinese folk medicine to alleviate pain symptoms, inflammatory disease, and different kinds of cancer^14–19^. In recent years, it has been demonstrated to be a highly effective analgesic and the analgesic properties of *Gelsemium* are likely conferred by alkaloids, which may thus have considerable potential as pharmaceuticals^16,20^. Koumine is one of the most abundant alkaloids found in *Gelsemium* ^21,22^. Based on our pilot pharmacological study, koumine has numerous potent biological properties. (1) Koumine reversed acetic acid-, formalin- and complete Freund’s adjuvant (CFA) -induced inflammatory pain as well as chronic constriction injury (CCI)- and L5 spinal nerve ligation (L5 SNL)-induced neuropathic pain in a dose-dependent manner^20^. (2) Koumine treatment of diabetic rats was more effective than gabapentin in decreasing neuropathic pain behavior^23^. (3) Repeated treatment of koumine significantly reduced the damage to axons and myelin sheaths of the sciatic nerve and increased sensory nerve conduction velocity after nerve injury^23^. (4) Koumine exhibited potent anxiolytic effects in classic rodent anxiety models, which could be significantly antagonized by strychnine, a glycine-receptor antagonist^24^. In addition, koumine is devoid of the troublesome adverse effects associated with traditional analgesic agents^19^. These studies all indicate that koumine has great potential as an effective therapeutic agent.

Interestingly, we previously demonstrated that increased AP levels in the SC appeared to mediate the analgesic effects of koumine on neuropathic pain^20^. Moreover, numerous studies have demonstrated that agents that modulate the neuroinflammation-glia-steroid network can reduce nociceptive transmission^25–30^. Therefore, we speculated that koumine administration might be a new therapeutic approach for POP treatment. We thus characterized the antinociceptive effects of koumine in the paw incision model of POP and profiled the pharmacologic basis of koumine’s analgesic characteristics using TSPO antagonist PK11195 and GABA_A_ receptor antagonist bicuculline. In order to elucidate the relationship between the analgesic effects of koumine on POP and the neuroinflammation-glia-steroid network in the SC, we performed an analysis of the effects of koumine treatment on glial activation and proinflammatory cytokines expression in the lumbar SC over a time course corresponding to the development and progression of POP-induced pain symptoms.

## Results

### Subcutaneous administration of koumine significantly reduced plantar incision- induced POP in rats

**Figure 1:**
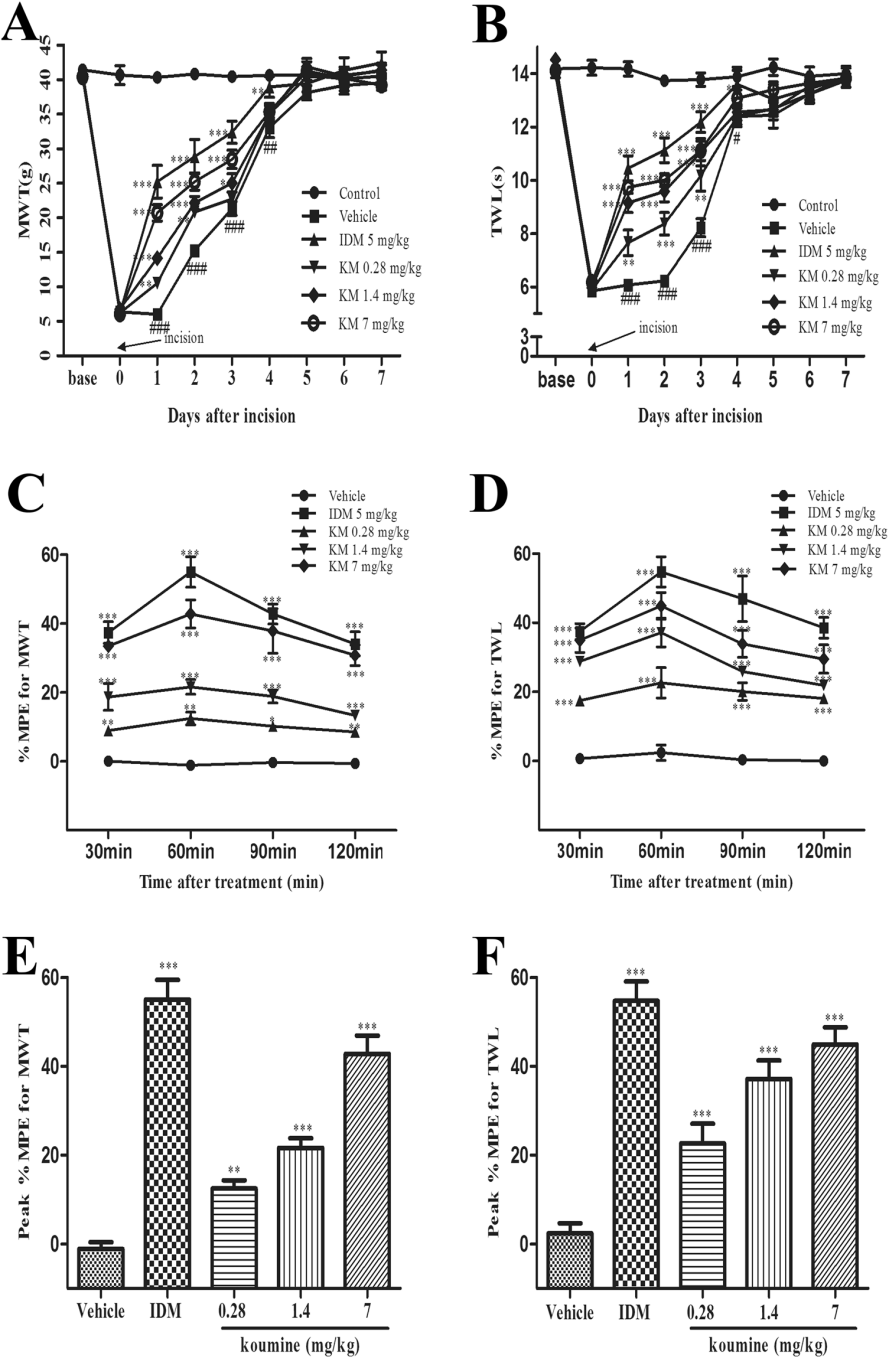
Subcutaneous treatment with koumine alleviated incision induced mechanical allodynia and thermal hyperalgesia. (**A**,**B**) Repeated koumine treatment attenuated the effects of plantar incision on pain as measured by MWT (**A**) and TWL (**B**) over time. (**C**,**D**) Maximal possible effects (%MPE) for MWT (**C**) and TWL (**D**) were increased by acute koumine administration in a dose dependent manner. (**E**,**F**) Similarly, koumine elevated the peak %MPE for MWT and TWL 1 h after treatment on the first day post incision. For (**A**,**B**), vehicle, koumine (0.28, 1.4, 7.0 mg/kg) or indomathacin (5 mg/kg) was administered once per day by s.c. injection for 7 consecutive days beginning at postoperative day 1. For (**C**–**F**), the drugs was administered s.c. on day 1 post incision. Abbreviations; KM: koumine, IDM: indomethacin. Data are presented as mean ± SEM. *P < 0.05, **P < 0.01, ***P < 0.001 versus vehicle group; ^#^ *P* < 0.05, ^##^ *P* < 0.01, ^###^ *P* < 0.001 versus control group. Repeated measures two-way ANOVA with time and treatment as main effect. For further comparison among groups at the same time, multivariate ANOVA was performed followed by the LSD test. Each group consisted of 5–11 rats.

In the present study, we aimed to measure the effects of subcutaneous (s.c.) administration of koumine on plantar incision-induced POP in rats. Notably, we have previously shown that there were no observable physiologic effects of koumine in untreated rats. We therefore induced POP using the plantar incision model and assessed pain in repeated koumine treated rats and controls by determining the effect of treatment on thermal withdrawal latency (TWL) and mechanical withdrawal threshold (MWT) (i.e. thermal and mechanical allodynia, respectively) over the first week post-surgery. We found that plantar incision significantly decreased the withdrawal threshold to thermal stimulation and mechanical stimulation and the development of thermal hyperalgesia and mechanical allodynia peaked on postoperative day 1 and then decreased gradually until approaching baseline on day 6–7 (Fig. 1 A and B). TWL and MWT measured in the hind paw ipsilateral to surgery demonstrated that repeated koumine treatment at a dose range between 0.28 to 7.0 mg/kg attenuated the intensity of persistent POP in a dose-dependent manner. Similar effects were confirmed for indomethacin (5 mg/kg) administration, which was used as a positive control.

To more fully characterize time course of acute analgesic effects of a single s.c. administration of koumine, we calculated maximal possible effect (%MPE) for MWT and TWL. As is shown in Fig. 1C and D, koumine significantly elevated %MPE for mechanical allodynia and thermal hyperalgesia dose-dependently. Moreover, the peak %MPE for both mechanical allodynia and thermal hyperalgesia was observed 1 h after koumine treatment (Fig. 1C and D) and was similarly found to be dose dependent (Fig. 1E and F).

### Intrathecal administration of koumine exerted analgesic effects on plantar incision-induced POP in rats

**Figure 2:**
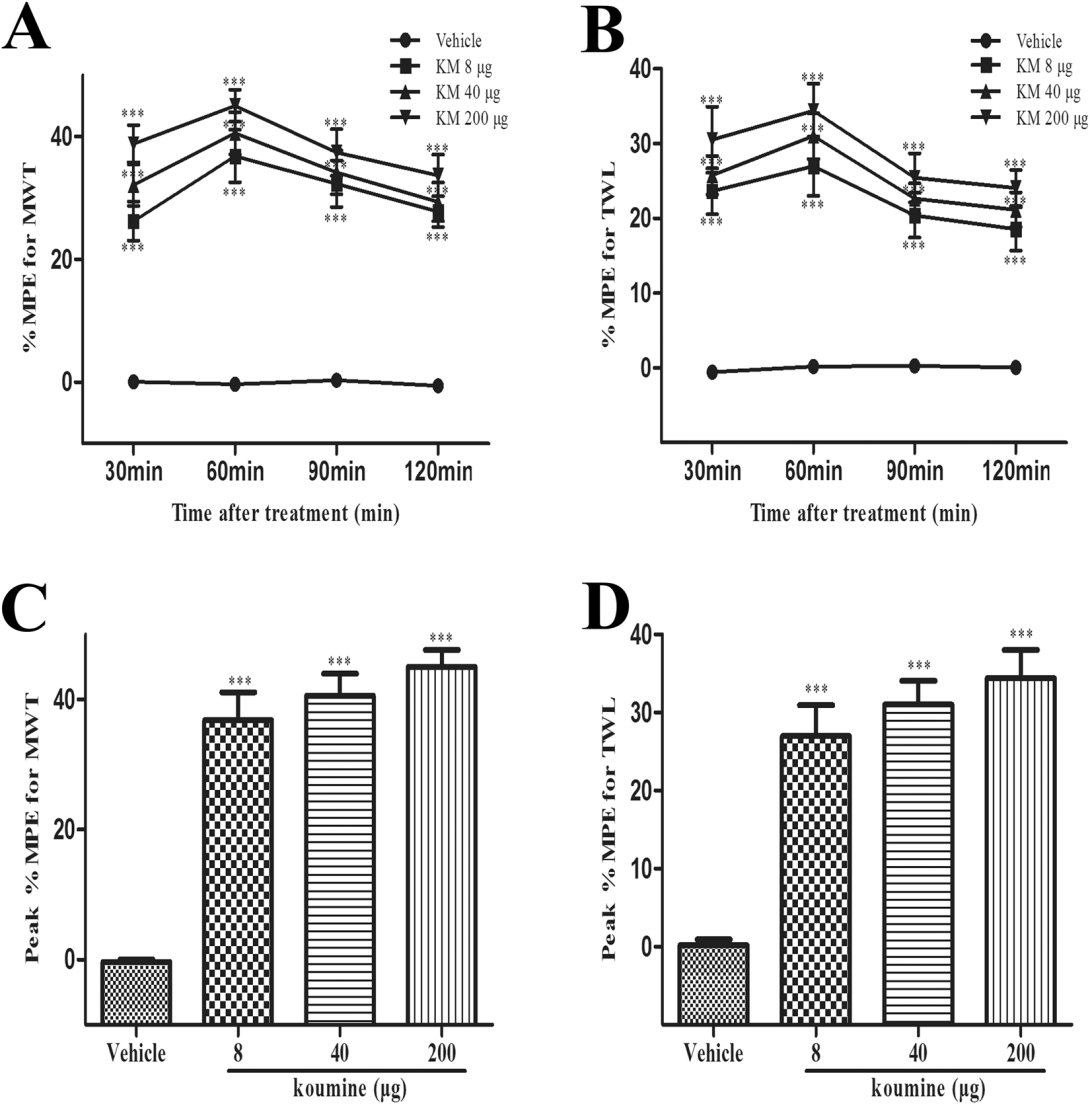
Intrathecal treatment with koumine alleviated incision induced mechanical allodynia and thermal hyperalgesia. (**A**,**B**): %MPE for MWT (**A**) and TWL (**B**) after a single i.t. injection of vehicle or koumine (8, 40 or 200 μg) on the first day post incision reveal an analgesic effect of koumine for at least 2 h post treatment. (**C**,**D**): The peak %MPE for MWT and TWL at 1 h after treatment on the first day post-incision. Abbreviations; KM: koumine. Data are presented as mean ± SEM. ***P < 0.001 versus vehicle group. Repeated measures two-way ANOVA with time and treatment as main effect. For further comparison among groups at the same time, multivariate ANOVA was performed followed by the LSD test. Each group consisted of 8–11 rats.

As we have found previously that koumine treatment led to significantly elevated AP levels in SC, we speculated the SC may be central to the analgesic functions of koumine. To test this hypothesis, we carried out intrathecal (i.t.) administration of koumine directly into the SC and assessed its analgesic activity. We found koumine significantly inhibited mechanical allodynia and thermal hyperalgesia over a range of doses (8–200 μg) over a time course lasting at least 2 h (Fig. 2A and B). Peak %MPE was observed 1 h after koumine treatment on the first day post-incision and was similarly found to be dose dependent (Fig. 2C and D). These data indicate that the analgesic effects of koumine were mediated at least in part by acting on the SC.

### Koumine significantly inhibited glial activation in the spinal dorsal horn of POP rats

**Figure 3:**
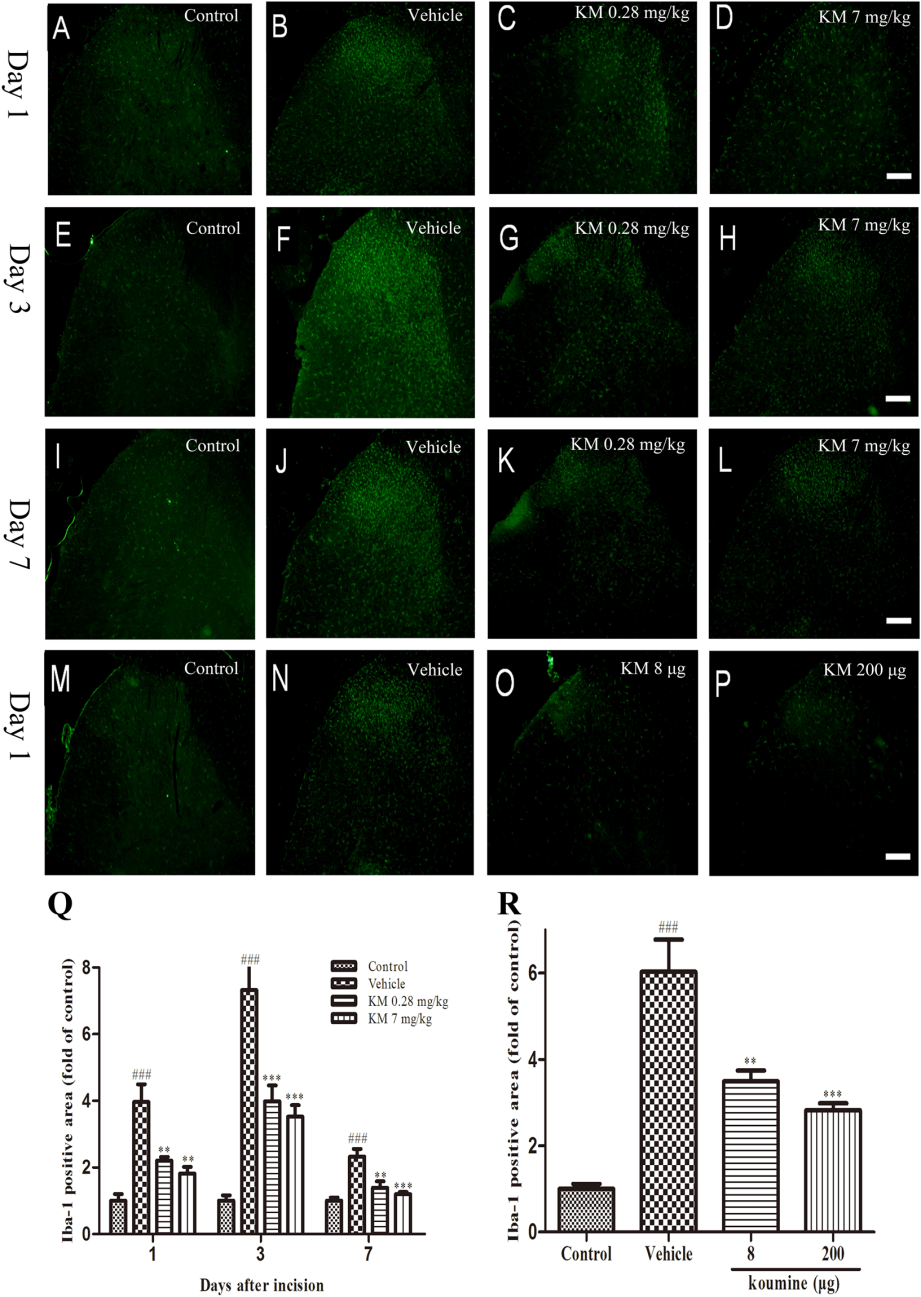
Koumine treatment attenuated POP-induced microglia activation in the ipsilateral spinal dorsal horn of rats. (**A**–**L**): Representative images of ipsilateral dorsal spinal cord microglia activation revealed by Iba-1 staining (green) in treatment and control groups receiving subcutaneous injections (as noted by the label on each image) at day 1 (**A**–**D**), day 3 (**E**–**H**), and day 7 (**I**–**L**) post-incision. (**Q**) Quantification of Iba-1 positive area in treatment and control groups receiving subcutaneous injections. (**M**–**P**) and (**R**) are representative images and quantification of ipsilateral dorsal spinal cord microglia activation in treatment and control groups receiving intrathecal injections, respectively. Scale bar: 100 μm. For (**A**–**L**), vehicle or koumine (0.28, 7 mg/kg) was administered once per day by s.c. injection for 1 (**A**–**D**), 3 (**E**–**H**), 7 (**I**–**L**) days beginning at postoperative day 1. For (**M**–**P**), vehicle or koumine (8, 200 μg) was administered by a single i.t. injection on the first day post incision. Abbreviations; KM: koumine. Data are presented as mean ± SEM. **P < 0.01, ***P < 0.001 versus vehicle group; ^###^ *P* < 0.001 versus control group. One-way ANOVA followed by the LSD post hoc test. Each group consisted of 5–11 rats.

**Figure 4:**
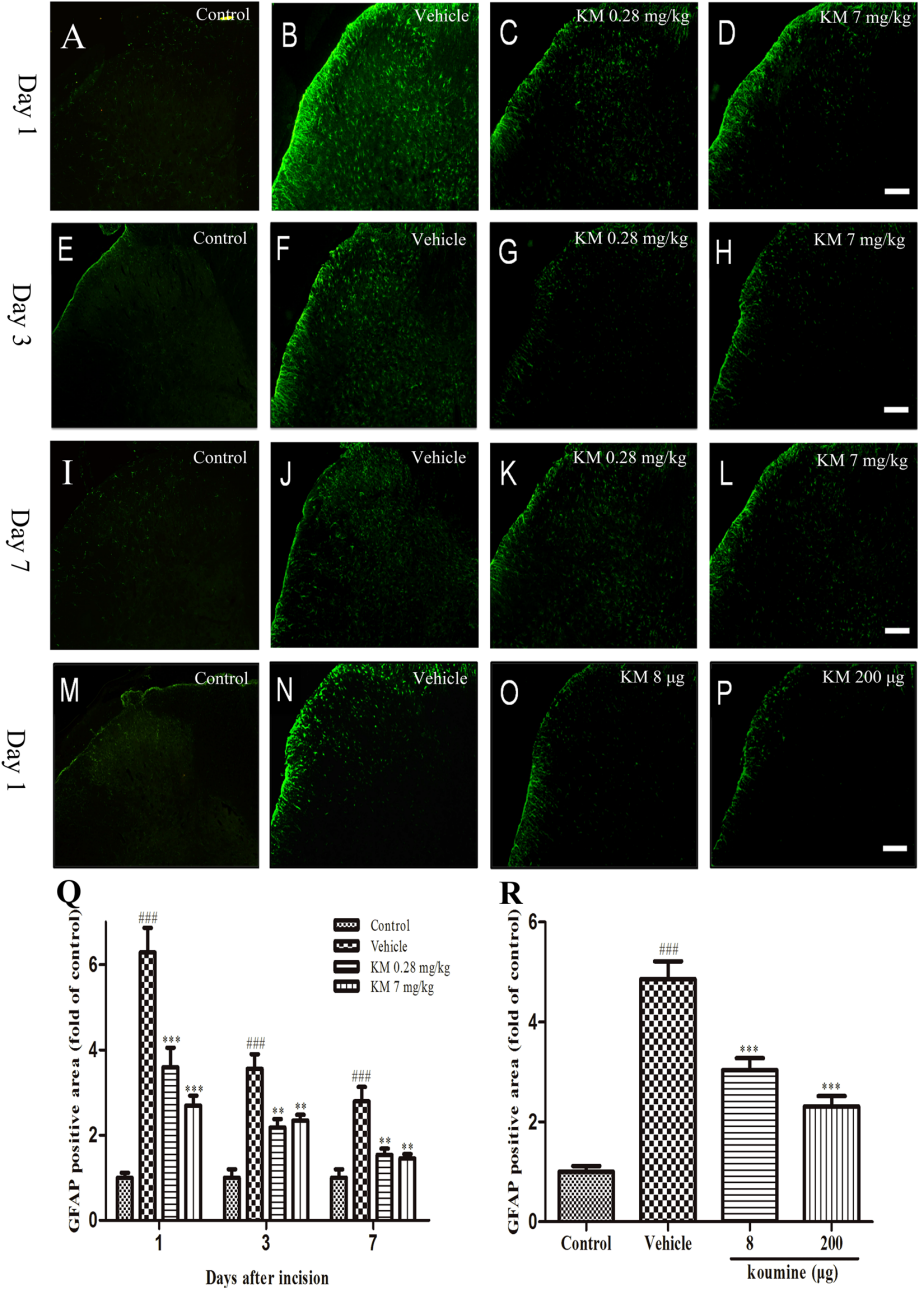
Koumine treatment attenuated POP-induced astroglia activation in the ipsilateral spinal dorsal horn of rats. (**A**–**L**) Representative images of ipsilateral dorsal spinal cord astroglial activation revealed by GFAP staining (green) in treatment and control groups receiving subcutaneous injections (as noted by the label on each image) at day 1 (**A**–**D**), day 3 (**E**–**H**), and day 7 (**I**–**L**) post-incision. (**Q**) Quantification of GFAP positive area in treatment and control groups receiving subcutaneous injections. (**M**–**P**) and (**R**) are representative images and quantification of ipsilateral dorsal spinal cord astroglial activation in treatment and control groups receiving intrathecal injections, respectively. Scale bar: 100 μm. For (**A**–**L**), vehicle or koumine (0.28, 7 mg/kg) was administered once per day by s.c. injection for 1 (**A**–**D**), 3 (**E**–**H**), 7 (**I**–**L**) days beginning at postoperative day 1. For (**M**–**P**), vehicle or koumine (8, 200 μg) was administered by a single i.t. injection on the first day post incision. Abbreviations; KM: koumine. Data are presented as mean ± SEM. **P < 0.01, ***P < 0.001 versus vehicle group; ^###^ *P* < 0.001 versus control group. One-way ANOVA followed by the LSD post hoc test. Each group consisted of 5–11 rats.

POP caused by central sensitization is characterized by a robust response of microglia and astrocytes referred to as gliosis^31^. We therefore set out to determine if koumine inhibited the activation of microglia and astrocytes in response to plantar incision. Glial activation can be assessed by the levels of immunoreactivity for ionized calcium binding adaptor protein-1 (Iba-1) and glial fibrillary acidic protein (GFAP), which label microglia and astroglia, respectively. We therefore carried out fluorescent immunohistochemistry for these markers in tissue from the dorsal horn of L4–L5 SC ipsilateral to plantar incision, as shown in Figs 3 and 4. We first quantified the fluorescence density for Iba-1 and determined that plantar incision significantly enhanced Iba-1 immunohistochemical staining density from post-operative day 1 to the end of the observation period. The most intense staining for Iba-1 was observed on day 3 (*P < *0.001). Indeed, daily s.c. administration of koumine (0.28 or 7.0 mg/kg) significantly inhibited Iba-1 immunohistochemical staining density across the experimental time course and the effects were most significant on day 3 post-incision (Fig. 3, A–L and Q). Moreover, a single i.t. injection of koumine (8 or 200 μg) similarly significantly inhibited the density of Iba-1 immunohistochemical staining on day 1 post-incision (Fig. 3, M–P and R).

We next determined the effect of koumine on astroglial activation and found a similar effect as we observed for microglia. Incision significantly enhanced the density of GFAP immunohistochemical staining, which peaked on day 1 post-incision and then decreased afterwards (Fig. 4, A–L, Q). Daily s.c. administration of koumine (0.28 or 7.0 mg/kg) significantly inhibited GFAP immunohistochemical staining across all time points, but most especially on day 1 post-incision (Fig. 4, A–L and Q). Moreover, a single i.t. injection of koumine (8 or 200 μg) also significantly attenuated the density of GFAP immunohistochemical staining at day 1 post-incision (Fig. 4, M–P and R).

### Koumine significantly downregulated proinflammatory cytokines in the SC of POP rats

**Figure 5:**
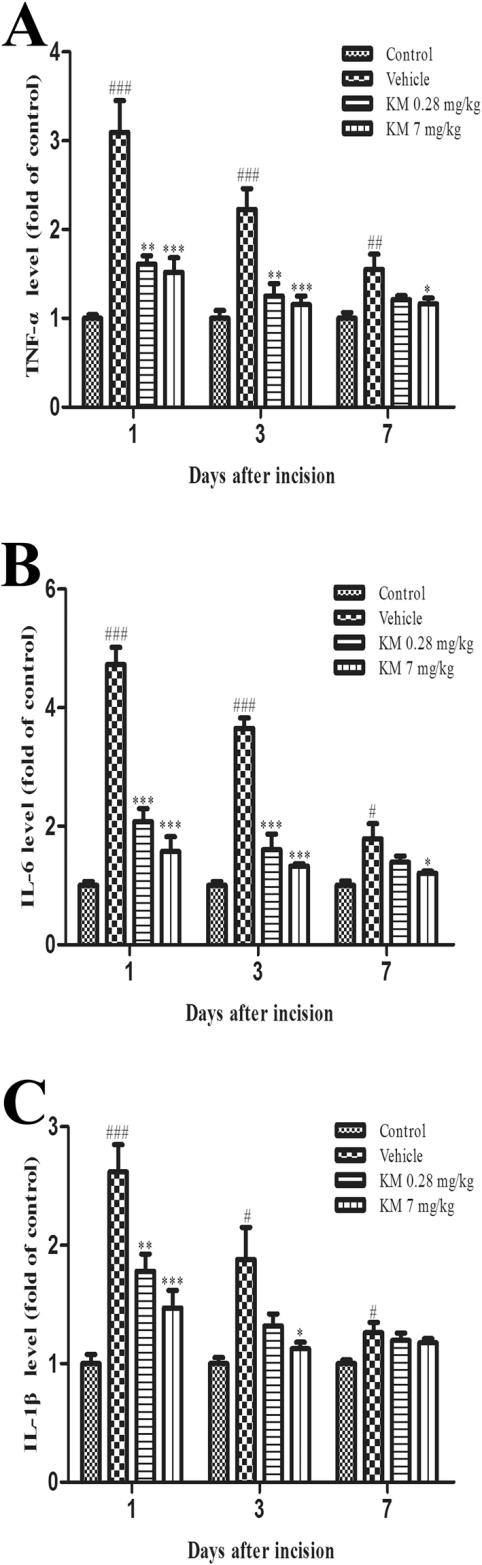
Koumine attenuated POP-induced proinflammatory cytokine overexpression in rat SC. Plantar incision led to significantly increased levels of TNF-α (**A**), IL-6 (**B**), and IL-1β (**C**). The peak increase of all cytokines was observed on day 1 post incision. Two different doses of koumine attenuated the rise in the levels of all 3 cytokines. For (**A**–**C**), vehicle or koumine (0.28, 7 mg/kg) was administered once per day by s.c. injection for 1, 3 or 7 days beginning at postoperative day 1. Abbreviations; KM: koumine. Data are presented as mean ± SEM. *P < 0.05, **P < 0.01, ***P < 0.001 versus vehicle group; ^#^ *P* < 0.05, ^##^ *P* < 0.01, ^###^ *P* < 0.001 versus control group. One-way ANOVA followed by the LSD post hoc test. Each group consisted of 5–11 rats.

Microglial and astroglial activation is associated with increases in proinflammatory cytokines, which can lead to damage in the CNS. We therefore tested the expression levels of cytokines known to be highly involved in neuroinflammation by ELISA after plantar incision. We observed significantly increased levels of tumor necrosis factor α (TNF-α), interleukin 1β (IL-1β), and interleukin 6 (IL-6) production in the SC 1 day after incision (Fig. 5). The observed levels were lower on day 3 and 7 post-incision than on day 1, but were still significantly higher than control. Daily s.c. administration of koumine (0.28 or 7.0 mg/kg) significantly attenuated the increases in TNF-α, IL-1β and IL-6 expression, especially on day 1 and 3 post-incision (Fig. 5).

### Antagonists of TSPO and GABA_A_ receptor inhibited koumine’s analgesic effects on POP in rats

**Figure 6:**
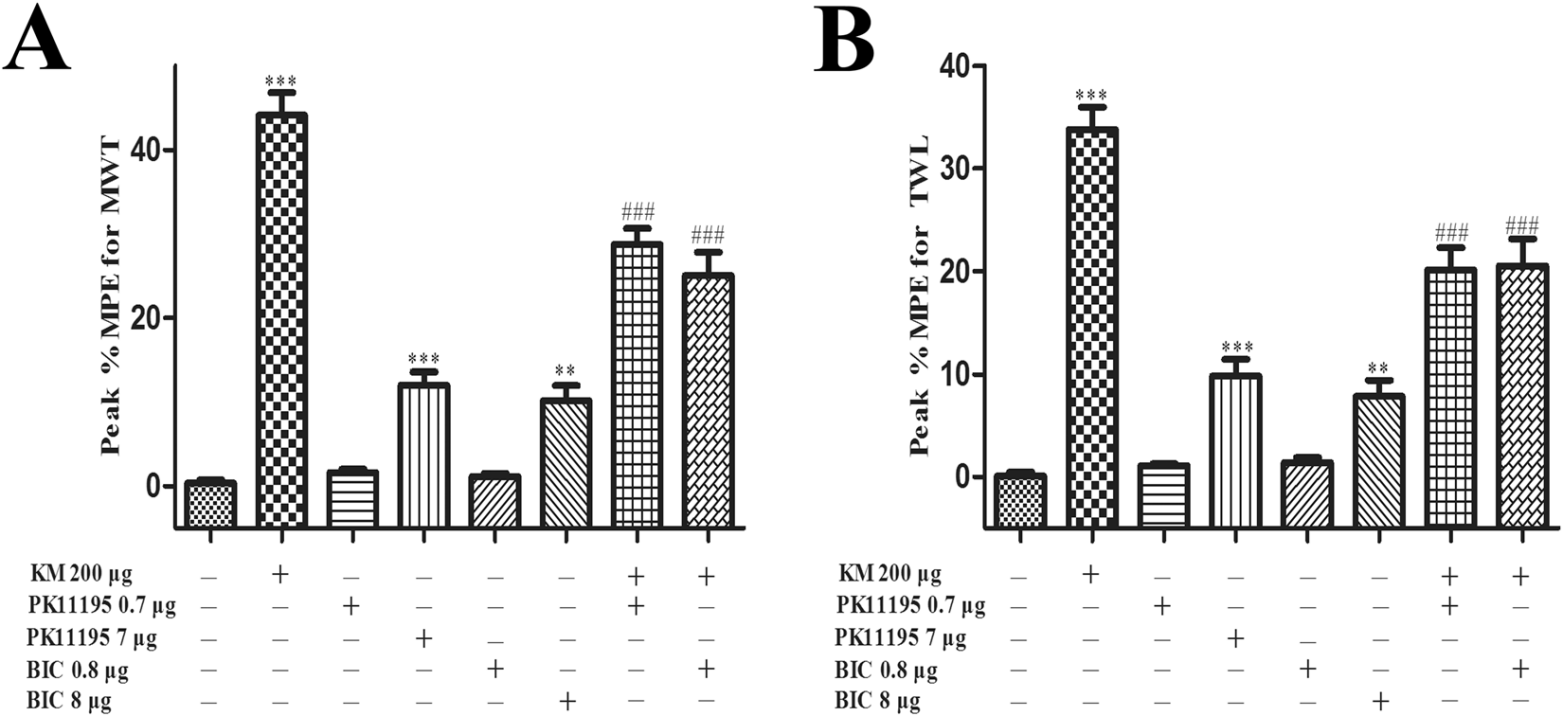
Antagonists of TSPO and GABA_A_ receptor inhibited koumine’s analgesic effects on POP in rats. Effect of koumine (200 μg), PK11195 (0.7, 7 μg), bicuculline (0.8, 8 μg) and their co-administration on peak %MPE for MWL (**A**) and TWL (**B**) 60 min after treatment on the first day post-incision, respectively. Koumine was injected intrathecally preceded by vehicle or various antagonists including PK11195 and bicuculline. Abbreviations; KM: koumine, BIC: bicuculline. Data are presented as mean ± SEM. **P < 0.01, ***P < 0.001 versus vehicle group, ^###^ *P* < 0.001 versus koumine alone group. One-way ANOVA followed by the LSD post hoc test. Each group consisted of 5–12 rats.

We next sought to elucidate the relationship between spinal analgesic effects of koumine and TSPO function, which mediates neurosteroid production. We therefore pretreated either the TSPO antagonist PK11195 or the GABA_A_ receptor antagonist bicuculline before the i.t. administration of koumine and assayed for the effect of each on the analgesic actions of koumine. As shown in Fig. 6A and B, analysis of peak %MPE revealed that i.t. injection of bicuculline and PK11195 partly reversed the analgesic effect of koumine on both %MPE for MWT and TWL. Moreover, treatment with either PK11195 or bicuculline alone can led to increases in peak %MPE for MWT and TWL at a dose of 7 μg and 8 μg, respectively. Taken together, these results indicate that koumine may exerted its pharmacologic effect through TSPO.

## Discussion

This study demonstrate that koumine, the main alkaloidal constituent of *Gelsemium elegans* Benth., attenuates pain behavior in a rat model of POP. As administration of koumine significantly inhibited microglia and astroglia activation as well as proinflammatory cytokines expression in the SC, koumine-induced anti-allodynic effects on POP can be mainly attributed to inhibitory effect on neuroinflammation. Pharmacologically, neurosteroid modulation in the SC might play an important role in mediating koumine’s analgesic effects, given the i.t. pretreatment of the TSPO antagonist PK11195 and GABA_A_ receptor antagonist bicuculline partly prevented i.t. koumine-induced analgesia.

*Gelsemium elegans* Benth has been reported to have diverse biological effects with therapeutic potential. However, the development of clinical applications has been restricted by *Gelsemium elegans* Benth.’s relatively high toxicity^22^. Currently, pharmacologists are trying to derive monomers with high potency and low toxicity from *Gelsemium elegans* Benth.. Previously, we developed a protocol that enabled us to obtain several different monomers from *Gelsemium elegans* Benth. tissue by a pH-zone–refining counter-current chromatography technique^21^. This subsequently allowed us to carry out pharmacodynamic screening of this panel of *Gelsemium elegans* Benth. derived compounds. Our preliminary experimental evidence indicated that gelsenicine, the most toxic alkaloid derived from *Gelsemium elegans* Benth., exerted analgesic activity against inflammatory and neuropathic pain^32^. Similarly, Zhang *et al*. recently reported that gelsemine, another main alkaloid from *Gelsemium sempervirens* Ait. displayed potent and specific antinociceptive properties in chronic pain^15^. We have previously found that the isolated *Gelsemium elegans* Benth. compounds koumine exhibits potent analgesic, anti-inflammatory and anxiolytic effects, and its toxicity is relatively low compared with other alkaloid extracts of *Gelsemium elegans* Benth^20,33^. This all suggests that koumine may have a large range of promising clinical applications.

In the present study, repeated s.c. administration of koumine reversed thermal hyperalgesia and mechanical allodynia in a dose-dependent manner in the POP model, indicating that koumine may be particularly effective in treating POP. Growing evidence has indicated that glial cells play an important role in POP. In our POP model we observed an increase in the expression of Iba-1 (microglial marker) and GFAP (astroglia marker) in the lumbar dorsal horn ipsilateral to paw incision. Microglial reactivity was enhanced from post-operative day 1 to the end of the observation period, the most intense staining for Iba-1 was observed on day 3. Astroglia reactivity was peaked on day 1 post-incision and then decreased afterwards, which is in accordance with a previous report that paw incision-induced GFAP and Iba-1 expression was associated with the initiation and maintenance of mechanical hypersensitivity^31^.

Moreover, glia are known to produce and release proinflammatory cytokines, including IL-1β, TNF-α, and IL-6, quite rapidly from dorsal SC after injury, which further stimulates glial cells (positive feedback) and neurons^34^. This upregulation of spinal proinflammatory cytokines in POP has been confirmed by our current study. Interestingly, in our study koumine was found to not only inhibit spinal dorsal horn astroglia and microglia activation throughout the entire POP process, but also to downregulate elevated spinal proinflammatory cytokines induced by paw incision. This indicates that koumine-induced antinociceptive effects for POP might possibly be attributed to the inhibition of neuroinflammation. As early microglial reactivity in POP is downstream of early astrocytic reactivity, which was inhibited by koumine, we speculate that koumine is more likely to firstly influence astroglia related factors and subsequently affect other neuroinflammation factors (like microglia).

Apart from neuroinflammation involving glia activation, koumine might also be able to regulate neurosteroid related functions in SC. Koumine’s dual neurosteroid and neuroinflammatory action may thus have a close interrelationship with each other. This is suggested by our previous finding that increased AP in the SC appeared to mediate the analgesic effect of koumine on neuropathic pain^20^. To further explore the relationship between neurosteroids and koumine’s analgesic effects, in the current study we i.t. administrated PK11195, a TSPO antagonist, and bicuculline, a GABA_A_ receptor antagonist, before i.t. treatment with koumine. Interestingly, we found that the antinociceptive activities of koumine were significantly prevented by both agents, suggesting that TSPO might be important targets of koumine’s analgesic function.

TSPO is a five transmembrane domain protein that is localized primarily in the outer mitochondrial membrane and is expressed predominantly in steroid-synthesizing tissues, including the SC and brain. TSPO facilitates the translocation of cholesterol from the outer to the inner mitochondrial membrane, which is the rate-limiting step for neurosteroidogenesis^35^. A growing number of evidence indicates that neurosteroids plays a vital role in pain states, such as AP^36–39^. It is known that the analgesic action of the AP is mediated by a direct positive allosteric modulation of GABA_A_ receptor in the SC of rats displaying mechanical or thermal pain symptoms^10^, and that exogenously administered AP-like compounds are particularly efficient for limiting pain symptoms^38,40^. In our study, koumine’s effects were also blunted by bicuculline, a GABA_A_ receptor antagonist. When considering these findings with our previous observation that koumine upregulated spinal AP, we speculate that koumine might act as a TSPO ligand (i.e. agonist) to stimulate AP generation and koumine exerted its antinociceptive action through AP, the GABA_A_ receptor modulator. It should be noted that bicuculline at high dosage (8 μg) alone exhibited analgesic effects toward POP, which might be associated with the inhibition of dorsal root reflexes (DRRs) in POP. Previous studies have demonstrated that bicuculline administered i.t. can effectively block DRRs and neurogenic inflammation^41^, although this function was not tested in POP model prior to our study.

TSPO is also a promising drug target for controlling neuroinflammation, although the exact mechanism is still unclear. In response to neuroinflammation, TSPO expression is significantly increased in microglia and astroglia in the CNS. Positron emission tomography imaging with radiotracers that target TSPO have been developed to assess neuroinflammatory processes. Indeed, TSPO is used as a biomarker of neuroinflammation^42,43^. Furthermore, TSPO ligands have been shown to reduce the activation of glia and inhibit inflammatory responses. For example, administration of the TSPO ligand etifoxine inhibited macrophages and glial activation, and reduced proinflammatory cytokines levels after traumatic brain injury^44^. The selective TSPO ligands Ro5-4864 and PK11195 have also been shown to inhibit the first- and second-phase responses in a formalin-induced inflammatory pain model^45^. In THP-1 and BMDM cells, Ro5-4864 potently suppressed ATP-induced pyrin domain containing 3 (NLRP3) inflammasome activation^46^. Therefore, it is possible that koumine functions as a TSPO ligand to inhibit neuroinflammation, as koumine treatment significantly reduced the spinal level of glial activation and the production of proinflammatory cytokines. It should be noted that, in present study we firstly found that high dose PK11195 (7 μg) also exerted analgesic effects, demonstrating the potent anti-inflammatory activity of TSPO ligands against POP status.

On the basis of our work and others^47–49^, we reason that a pronociceptive and antinociceptive mechanism may coexist in the state of pain. The former includes, but not limited to, prostaglandin, histamine and proinflammatory cytokines that play important roles in the occurrence and development of pain. Pain can also lead to the production of antinociceptive molecules such as endocannabinoids and neurosteroids, which may represent an adaptive response to pain and elicit beneficial effects against a diverse range of pathological pain symptoms. Studies have shown that TSPO agonist such as etifoxine can inhibit glial activation and reduce proinflammatory cytokines levels after traumatic brain injury^44^. In mononeuropathy, etifoxine can also stimulate allopregnanolone synthesis in the SC to produce analgesic^36^. Therefore, we speculate that koumine may function as a TSPO agonist to inhibit microglial and astroglial activation, suppress expression of proinflammatory cytokines, and meanwhile stimulate allopregnanolone synthesis to produce analgesia. In addition, several studies have shown that AP may control the expression of inflammatory cytokines through a decay-accelerating factor (DAF, CD55)-regulated mechanism^50,51^. It is conceivable that AP may directly inhibit inflammatory cytokines release in POP as well. Whether TSPO is the target of koumine and whether koumine acts through AP to exert its antinflammatory effects remain open questions and merit further investigation.

In conclusion, koumine prevented mechanical allodynia and thermal hyperalgesia caused by POP. The pharmacologic mechanism of koumine-mediated analgesia might involve inhibition of spinal neuroinflammation and activation of TSPO mediated analgesic effect. These data suggested that koumine might be a potential pharmacotherapy for the management of POP.

## Materials and Methods

### Animals

Male adult Sprague-Dawley rats weighing of 180 to 200 g (Shanghai Laboratory Animal Center at the Chinese Academy of Sciences, Shanghai, China) were housed in a temperature-controlled room (25 ± 2 °C) on a 12-h light/dark cycle (lights on 08:00 AM), with free access to standard laboratory food and water, except during behavioral observations. Rats were housed for at least 1-week before undergoing experiments. Rats were assigned to one behavioral experiment, and experiments were performed between 09:00 and 17:00. All rat experiments were performed in accordance with the National Institutes of Health Guide for Care and Use of Laboratory Animals (Publication No. 85-23, revised 1985) and were approved by the Committee of Ethics of the Fujian Medical University (Fujian, China). All procedures complied with the guidelines for animal care and use established at the Fujian Medical University.

### Chemicals and reagents

Koumine (molecular formula, C_20_H_22_N_2_O; molecular weight, 306.1804; CAS registry number, 1358-76-5) of 99% purity was isolated from *Gelsemium elegans* Benth. by pH-zone–refining counter-current chromatography as described previously^21^. indomethacin (Shanghai Xinyi Jiufu Pharmaceutical Co., Ltd, Shanghai, China) were used for positive control. Rabbit anti-ionized calcium binding adaptor molecule 1 antibody (anti-Iba-1) and rabbit anti-glial fibrillary acidic protein antibody (anti-GFAP) were purchased from Abcam (Cambridge, UK). Fluorescein (FITC)-conjugated goat anti-rabbit was supplied by Jackson Immuno Research (West Grove, PA, USA). Normal rabbit and goat serum were purchased from Biosynthesis Biotechnology. 1-(2-Chlorophenyl)-N-methyl-N-(1-methylprolyl)-3-isoquinoline carboxamide, (PK11195, Sigma-Aldrich, St. Louis, MO, USA) and bicuculline (Sigma-Aldrich, St. Louis, MO, USA) were pharmaceutical grade. All other reagents used were analytical grade. Koumine and indomethacin was prepared daily prior to use in sterile physiological saline (0.9% w/v sodium chloride), and administered by subcutaneous (s.c.) injection at a dose of 4 ml/kg rat body weight.

### POP model by rat plantar incision

Plantar incisional surgery was performed as previously described^4^. Rats were anesthetized with isoflurane (2%) via a nose mask. The plantar surface of the right hindpaw was prepared in a sterile manner with a 10% povidone-iodine solution. A longitudinal 1 cm incision was made through the skin and fascia, starting 0.5 cm from the proximal edge of the heel and extending toward the toes of the right hindpaw. The plantaris muscle was elevated and incised longitudinally, leaving the muscle origin and insertion intact. After hemostasis with gentle pressure, the skin was sutured with 2 mattress sutures of 5-0 nylon.

### Measurement of thermal hyperalgesia and mechanical allodynia in rats

Thermal hyperalgesia was determined using a commercial thermal paw stimulator (PL-200, Chengdu Technology & Market Co, Ltd, Sichuan, China) as described by Hargreaves *et al*.^52^. In a temperature-controlled room (25 ± 2 °C), rats were placed into individual plastic cubicles mounted on a glass surface. The plantar surface of each hind paw was then exposed to a thermal stimulus in the form of radiant heat emitted from a focused projection bulb for a maximal exposure time of 16-sec to minimize possible tissue damage. The procedure was repeated twice at 10 min intervals and paw TWL was calculated as the mean of the 2 latencies. Mechanical allodynia was determined using a commercial electronic von Frey apparatus (Model 2390; IITC Life Science Inc., Woodland Hills, CA, USA) as described by Mitrirattanakul *et al*.^53^, but with minor modifications. Rats were placed into a Plexiglas box on a steel mesh floor. The center of the hind paw was stimulated using the von Frey filament applied up to a maximum strength of 55 g or until the point of paw withdrawal. The threshold at which withdrawal occurred was automatically registered. The procedure was performed twice for each hind paw at 10 min intervals. MWT was calculated as the mean of the 2 values. For the measurement of acute effects of koumine, the vehicle, koumine (0.28, 1.4 or 7.0 mg/kg) and indomethacin (5 mg/kg) was administered s.c. on day1 post surgery. After treatment, the TWL and WMT were measured every 30 min for 2 h. For the repeated treatment study, rats were assigned to receive the drug once per day by s.c. injection for 7 consecutive days beginning at postoperative day 1, and the TWL and WMT were measured before surgery (baseline), before drug treatment (pre-dosing), and at different times after drug administration (post-dosing) on the morning of postoperative day 1–7.

### Immunofluorescence

Rats were anesthetized by intraperitoneal injection of 400 mg/kg chloral hydrate 1 h after drug administration on the morning of postoperative day 1, 3, and 7. The lumbar segments (L4–L5) of the SC were excised for the purpose of analysis by fluorescent immunohistochemistry as described previously, but with minor modifications^54^. Briefly, 300 ml of 0.9% saline was perfused transcardially. Then, 450 ml of 4% paraformaldehyde in 0.1 M phosphate buffer (PH 7.2–7.4, 4 °C) was perfused. The SC located between L4 and L5 was rapidly dissected and postfixed in the same fixative for 24 h. Tissues were immersed in 15% sucrose-containing PBS for 12 h and then transferred into 30% sucrose-containing PBS for 24 h. Tissues were then placed in Tissue-Tek^®^ OCT embedding medium (Sakura, Torrance, CA, USA) and immediately frozen at −22 °C. A Microm HM 525E cryostat (Francheville, France) was used to cut 16-μm–thick coronal sections that were subsequently mounted on glass slides coated with gelatin and chromium potassium sulfate. SC sections were preincubated for 1 h with the following sera in preparation for subsequent immunohistochemical experiments. For mono-labeling with anti-Iba-1 or anti-GFAP, SC sections were preincubated with 10% non-immune goat serum prepared in PB containing 0.3% Triton X-100 (PBT). Mono-labeled immunohistochemical experiments were conducted by incubating SC sections for 24 h at 4 °C with a primary antibody (anti-Iba-1, 1: 800 dilution; anti-GFAP, 1: 800 dilution) prepared in PBT. After being washed 3 times in PBS (5 min per rinse), sections were transferred into a solution containing the secondary antibody for 1 h at room temperature. Labeling solutions contained FITC-conjugated goat anti-rabbit prepared in PBT with a dilution ratio of 1: 800. After rinsing 3 times in PBS (5 min per rinse), sections were mounted with antifade mounting medium (Beyotime, Haimen China), and imaged under a fluorescence DMR microscope equipped with a digital camera (IX71-A12FL/PH, Olympus, Tokyo, Japan) assisted by a Pentium 4 PC. Fluorescence density was analyzed using Image-Pro Plus software (Media Cybernetics, Version 6.0).

### Intrathecal catheter insertion and drug administration

Intrathecal implantation of polyethylene tubing (Intramedic PE-10, Clay Adams, Parsippany, NJ, USA) into the subarachnoid space of the lumbar enlargement was performed in rats as described previously^55^. This method permits the direct administration of the drugs of interest to the SC. After 1 day of recovery, rats considered neurologically normal received 2% lidocaine (20 μl) through the intrathecal catheter to confirm postsurgical placement of the PE tubing within the subarachnoid space. Rats considered to be neurologically normal that displayed complete paralysis of both hind limbs and the tail after administration of lidocaine were used for the subsequent experiments. After 5 days recovery from intrathecal catheter implantation surgery, POP was induced by plantar incision. Effect of koumine (200 μg), PK11195 (0.7, 7 μg), bicuculline (0.8, 8 μg) and their co-administration on peak %MPE for MWL and TWL was tested on the first day post-incision, koumine was injected intrathecally preceded by vehicle or various antagonists including PK11195 and bicuculline. PK11195 were dissolved in 20% dimethyl sulfoxide (DMSO) and bicuculline were dissolved in 0.9% physiological saline, animals in vehicle group received 20% DMSO. The drugs were injected intrathecally in a volume of 5 μl followed by a 10 μl normal saline flush. MWT and TWL of the hind paws was measured 60 min after completion of the drug administration protocol. Visual confirmation of the placement of the PE tubing in the intrathecal space at the lumbar enlargement was performed by exposing the lumbar SC at the end of the each experiment. Data generated from rats with incorrect PE tubing position were excluded from the study.

### ELISA measurement of proinflammatory cytokines

For the measurement of cytokines levels, the lumbar segments (L4-L5) was rapidly dissected and homogenized in 1 ml PBS containing protease inhibitors (Complete protease inhibitor tablets, Roche). The concentrations of IL-6, IL-1β, and TNF-α were assayed using corresponding ELISA kits (R&D Systems). All assays were carried out in duplicate using recommended buffers, diluents and substrates. According to the manufacturer’s instructions, the absorbance was determined at 450 nm (Thermo Scientific, Multiskan FC Microplate Photometer) and the standard curve was included in each experiment. The concentration of the cytokines in the tissue was reported as pg/100 mg wet tissue.

### Statistical analysis

The analgesic effect of koumine was evaluated by the increment of the withdrawal threshold or latency after treatment and expressed as percentage of maximal possible effect (%MPE)^56^: %MPE = 100 × (WMT or TWL _post-treatment_ − WMT or TWL_post-incision_)/(WMT or TWL _pre-incision_ − WMT or TWL_post-incision_). Continuous data were expressed as means ± S.E.M. unless otherwise indicated. The data of behavioral tests were analyzed using repeated measures two-way ANOVA with time and treatment as main effect. For further comparison among groups at the same time, multivariate ANOVA was performed followed by the least significant difference test (LSD-t). For the data of immunochemistry and ELISA were tested using one-way ANOVA followed by the LSD post hoc test. Differences were considered statistically significant when *P* < 0.05. Statistical analyses were performed with SPSS (version 19.0, SPSS Inc., Chicago, IL, USA).

## References

[1] Chou R (2016). Management of Postoperative Pain: A Clinical Practice Guideline From the American Pain Society, the American Society of Regional Anesthesia and Pain Medicine, and the American Society of Anesthesiologists’ Committee on Regional Anesthesia, Executive Committee, and Administrative Council. J Pain.

[2] Gan TJ, Habib AS, Miller TE, White W, Apfelbaum JL (2014). Incidence, patient satisfaction, and perceptions of post-surgical pain: results from a US national survey. Curr Med Res Opin.

[3] Deumens R (2013). Prevention of chronic postoperative pain: cellular, molecular, and clinical insights for mechanism-based treatment approaches. Prog Neurobiol.

[4] Brennan TJ, Vandermeulen EP, Gebhart GF (1996). Characterization of a rat model of incisional pain. Pain.

[5] Duarte AM (2005). Reduction of postincisional allodynia by subcutaneous bupivacaine: findings with a new model in the hairy skin of the rat. Anesthesiology.

[6] Pogatzki EM, Niemeier JS, Brennan TJ (2002). Persistent secondary hyperalgesia after gastrocnemius incision in the rat. Eur J Pain.

[7] Obata H, Eisenach JC, Hussain H, Bynum T, Vincler M (2006). Spinal glial activation contributes to postoperative mechanical hypersensitivity in the rat. J Pain.

[8] Poisbeau P (2005). Inflammatory pain upregulates spinal inhibition via endogenous neurosteroid production. J Neurosci.

[9] Lacapère J-J, Papadopoulos V (2003). Peripheral-type benzodiazepine receptor: structure and function of a cholesterol-binding protein in steroid and bile acid biosynthesis. Steroids.

[10] Charlet A, Lasbennes F, Darbon P, Poisbeau P (2008). Fast non-genomic effects of progesterone-derived neurosteroids on nociceptive thresholds and pain symptoms. Pain.

[11] Romero A, Romero-Alejo E, Vasconcelos N, Puig MM (2013). Glial cell activation in the spinal cord and dorsal root ganglia induced by surgery in mice. Eur J Pharmacol.

[12] Melcangi RC, Panzica GC (2014). Allopregnanolone: state of the art. Prog Neurobiol.

[13] Shavit Y, Fridel K, Beilin B (2006). Postoperative pain management and proinflammatory cytokines: animal and human studies. J Neuroimmune Pharmacol.

[14] Yang J (2016). Effects of Koumine on Adjuvant- and Collagen-Induced Arthritis in Rats. J Nat Prod.

[15] Zhang J-Y, Gong N, Huang J-L, Guo L-C, Wang Y-X (2013). Gelsemine, a principal alkaloid from Gelsemium sempervirens Ait., exhibits potent and specific antinociception in chronic pain by acting at spinal α3 glycine receptors. Pain.

[16] Chen Z (1984). Extraction of Gelsemium alkaloids and the preliminary clinical research. J Navy Med.

[17] Chen Z (1986). Extraction and preliminary clinical application of alkaloids from Gelsemium. J Navy Med.

[18] Zhang L, Lin J, Wu Z (2003). Progression of chemical composition and phamacology on Gelsemium alkaloids. J Chin Med Mater.

[19] LinZhong TJQCZ (1988). Analgesic Effect and No Physical Dependence of Gelsemiumelegans Benth [J]. Pharmacol Clin Chin Mater Med.

[20] Xu Y (2012). Effects of koumine, an alkaloid of Gelsemium elegans Benth., on inflammatory and neuropathic pain models and possible mechanism with allopregnanolone. Pharmacol Biochem Behav.

[21] Su YP, Shen J, Xu Y, Zheng M, Yu CX (2011). Preparative separation of alkaloids from Gelsemium elegans Benth. using pH-zone-refining counter-current chromatography. J Chromatogr A.

[22] Jin GL (2014). Medicinal plants of the genus Gelsemium (Gelsemiaceae, Gentianales)–a review of their phytochemistry, pharmacology, toxicology and traditional use. J Ethnopharmacol.

[23] Ling Q (2014). Anti-allodynic and neuroprotective effects of koumine, a Benth alkaloid, in a rat model of diabetic neuropathy. Biol Pharm Bull.

[24] Liu M (2013). The active alkaloids of Gelsemium elegans Benth. are potent anxiolytics. Psychopharmacology (Berl).

[25] Romero‐Alejo E, Puig MM, Romero A (2016). Antihyperalgesic effects of dexketoprofen and tramadol in a model of postoperative pain in mice–effects on glial cell activation. J Pharm Pharmacol.

[26] Lin L (2016). Acupuncture-induced analgesia: the role of microglial inhibition. Cell Transplant.

[27] Liu X (2014). Early repeated administration of progesterone improves the recovery of neuropathic pain and modulates spinal 18kDa-translocator protein (TSPO) expression. J Steroid Biochem Mol Biol.

[28] Aouad M, Charlet A, Rodeau JL, Poisbeau P (2009). Reduction and prevention of vincristine-induced neuropathic pain symptoms by the non-benzodiazepine anxiolytic etifoxine are mediated by 3alpha-reduced neurosteroids. Pain.

[29] Sun Y (2014). Intrathecal injection of JWH015 attenuates remifentanil-induced postoperative hyperalgesia by inhibiting activation of spinal glia in a rat model. Anesth Analg.

[30] Zychowska M, Rojewska E, Makuch W, Przewlocka B, Mika J (2015). The influence of microglia activation on the efficacy of amitriptyline, doxepin, milnacipran, venlafaxine and fluoxetine in a rat model of neuropathic pain. Eur J Pharmacol.

[31] Romero-Sandoval A, Chai N, Nutile-McMenemy N, Deleo JA (2008). A comparison of spinal Iba1 and GFAP expression in rodent models of acute and chronic pain. Brain Res.

[32] Liu M (2011). Gelsenicine from Gelsemium elegans attenuates neuropathic and inflammatory pain in mice. Biol Pharm Bull.

[33] Liu H, Xu Y, Shi D, Yu C (2008). Pharmacognostical study on the Gelsemium elegans Benth. from Fuzhou. Strait Pharm J.

[34] Grace PM, Hutchinson MR, Maier SF, Watkins LR (2014). Pathological pain and the neuroimmune interface. Nat Rev Immunol.

[35] Rupprecht R (2010). Translocator protein (18 kDa) (TSPO) as a therapeutic target for neurological and psychiatric disorders. Nat Rev Drug Discov.

[36] Aouad M, Petit-Demouliere N, Goumon Y, Poisbeau P (2014). Etifoxine stimulates allopregnanolone synthesis in the spinal cord to produce analgesia in experimental mononeuropathy. Eur J Pain.

[37] Poisbeau P (2014). Analgesic strategies aimed at stimulating the endogenous production of allopregnanolone. Front Cell Neurosci.

[38] Meyer L, Patte-Mensah C, Taleb O, Mensah-Nyagan AG (2011). Allopregnanolone prevents and suppresses oxaliplatin-evoked painful neuropathy: multi-parametric assessment and direct evidence. Pain.

[39] Patte-Mensah C, Meyer L, Taleb O, Mensah-Nyagan AG (2014). Potential role of allopregnanolone for a safe and effective therapy of neuropathic pain. Prog Neurobiol.

[40] Meyer L, Venard C, Schaeffer V, Patte-Mensah C, Mensah-Nyagan AG (2008). The biological activity of 3alpha-hydroxysteroid oxido-reductase in the spinal cord regulates thermal and mechanical pain thresholds after sciatic nerve injury. Neurobiol Dis.

[41] Lin Q, Li D, Xu X, Zou X, Fang L (2007). Roles of TRPV1 and neuropeptidergic receptors in dorsal root reflex-mediated neurogenic inflammation induced by intradermal injection of capsaicin. Mol Pain.

[42] Notter T (2017). Translational evaluation of translocator protein as a marker of neuroinflammation in schizophrenia. Mol Psychiatry.

[43] Alam, M. M., Lee, J. & Lee, S.-Y. Recent Progress in the Development of TSPO PET Ligands for Neuroinflammation Imaging in Neurological Diseases. *Nucl Med Mol Imaging* 1–14 (2017). 10.1007/s13139-017-0475-8 PMC5721086 29242722

[44] Simon-O’Brien E, Gauthier D, Riban V, Verleye M (2016). Etifoxine improves sensorimotor deficits and reduces glial activation, neuronal degeneration, and neuroinflammation in a rat model of traumatic brain injury. J Neuroinflammation.

[45] DalBo S, Nardi GM, Ferrara P, Ribeiro-do-Valle RM, Farges RC (2004). Antinociceptive effects of peripheral benzodiazepine receptors. Pharmacol.

[46] Lee JW (2016). A translocator protein 18 kDa ligand, Ro5-4864, inhibits ATP-induced NLRP3 inflammasome activation. Biochem Biophys Res Commun.

[47] Krustev E, Rioux D, McDougall J (2015). Mechanisms and Mediators That Drive Arthritis Pain. Curr Osteoporos Rep.

[48] Tracey I, Dunckley P (2004). Importance of anti- and pro-nociceptive mechanisms in human disease. Gut.

[49] Coulombe M, Spooner M, Gaumond I, Carrier J, Marchand S (2011). Estrogen receptors beta and alpha have specific pro- and anti-nociceptive actions. Neurosci.

[50] He J, Evans C, Hoffman S, Oyesiku N, Stein D (2004). Progesterone and allopregnanolone reduce inflammatory cytokines after traumatic brain injury. Exp. Neurol..

[51] VanLandingham JW, Cekic M, Cutler S, Hoffman SW, Stein DG (2007). Neurosteroids reduce inflammation after TBI through CD55 induction. Neurosci Lett.

[52] Hargreaves K, Dubner R, Brown F, Flores C, Joris J (1988). A new and sensitive method for measuring thermal nociception in cutaneous hyperalgesia. Pain.

[53] Mitrirattanakul S (2006). Site-specific increases in peripheral cannabinoid receptors and their endogenous ligands in a model of neuropathic pain. Pain.

[54] Patte-Mensah C, Meyer L, Schaeffer V, Mensah-Nyagan AG (2010). Selective regulation of 3 alpha-hydroxysteroid oxido-reductase expression in dorsal root ganglion neurons: a possible mechanism to cope with peripheral nerve injury-induced chronic pain. Pain.

[55] Storkson RV, Kjorsvik A, Tjolsen A, Hole K (1996). Lumbar catheterization of the spinal subarachnoid space in the rat. J Neurosci Methods.

[56] Cheng JK, Chou RC, Hwang LL, Chiou LC (2003). Antiallodynic effects of intrathecal orexins in a rat model of postoperative pain. J Pharmacol Exp Ther.

